# Undiagnosed Hypertension in a Workplace: The Case of a Logistics Company in Gauteng, South Africa

**DOI:** 10.3390/healthcare9080964

**Published:** 2021-07-30

**Authors:** Morongwa Bokaba, Perpetua Modjadji, Kebogile Elizabeth Mokwena

**Affiliations:** Department of Public Health, School of Health Care Sciences, Sefako Makgatho Health Sciences University, P.O. Box 215, Ga-Rankuwa MEDUNSA, Pretoria 0204, South Africa; Morongs28@gmail.com (M.B.); kebogile.mokwena@smu.ac.za (K.E.M.)

**Keywords:** undiagnosed hypertension, risk factors, workplace, employees, logistic company, South Africa

## Abstract

A large proportion of the population with hypertension remains undiagnosed, untreated, or inadequately treated, contributing to the rising burden of cardiovascular diseases in South Africa. A workplace may either mitigate or accentuate the risk factors for hypertension. A cross sectional study was conducted to determine the prevalence of undiagnosed hypertension and associated factors among 312 employees in a Logistics Company, South Africa. A modified, validated, self-administered WHO STEPwise questionnaire was used to collect data on demography, lifestyle factors, anthropometry and blood pressure (BP). Hypertension was defined at BP ≥ 140/90 mmHg. Data was analysed using STATA 14. Mean age of employees was 40 ± 10 years, with a 50% prevalence of undiagnosed hypertension. No significant association was observed between occupation and undiagnosed hypertension, except for high prevalence of undiagnosed hypertension among truck drivers and van assistants (43%), and general workers (27%), having higher odds of increased waist-to-height ratio. Hypertension was associated with age (OR = 2.3, 95%CI; 1.21–4.27), alcohol use (AOR = 1.8, 95%CI; 1.05–2.93), waist circumference (AOR = 2.3, 95%CI; 1.29–4.07) and waist-to-height-ratio (AOR = 3.7, 95%CI; 1.85–7.30). Improved and effective workplace health programs and policies are necessary for management of undiagnosed hypertension among employees. Longitudinal studies on mediation of occupation in association of demographic and lifestyle factors with hypertension in workplaces are needed.

## 1. Introduction

The burden of hypertension has shifted from high-income countries (HICs) to low-and middle-income countries (LMICs), including sub-Saharan Africa (SSA) [1]. For the past few decades, SSA has been experiencing an exponential increase in hypertension with an estimated prevalence of 5% to 50% [2,3,4,5]. However, a large proportion of the population with hypertension remains undiagnosed, untreated, or inadequately treated, contributing to the rising burden of cardiovascular diseases (CVDs) in the region [5,6]. Failure to treat and control hypertension predisposes to higher risk of cardiovascular events, kidney diseases [7,8], stroke [9], metabolic syndrome, hypertensive retinopathy [10] and dementia [11].

South Africa has documented a health transition characterized by a quadruple burden of communicable, non-communicable diseases (NCDs), perinatal and maternal, and injury-related disorders, over a decade ago [12]. Ever since, the focus has been on NCDs emerging in both rural and urban areas, resulting in increasing pressure on acute and chronic health-care services, as one of the major public health concerns [12]. The country continues to record a higher mortality rate for NCDs than those for HIV/AIDS and tuberculosis combined [13,14], with CVDs being the leading category of NCDs [15]. Local studies in South Africa continue to report higher prevalence of hypertension (21% to 52%), in line with the epidemiological transition and increasing burden of chronic diseases of lifestyle [16,17,18,19,20]. The South Africa National Health and Nutrition Examination Survey (SANHANES) has reported that among those with hypertension, 48.7% were unscreened, 23.1% were screened but undiagnosed, 5.8% were diagnosed but untreated, 13.5% were treated but uncontrolled and 8.9% were controlled, suggesting that 91.1% of the hypertensive population has an unmet need for care [21].

The South African Medical Research Council (SAMRC) technical report on chronic diseases [22] has suggested a conceptual framework that links NCDs and risk factors, as well as risky behaviors. Four modifiable risky behaviors that NCDs are attributable to are tobacco smoking, unhealthy diet, physical inactivity and alcohol use [23,24]. These behaviors contribute to several risk factors, such as hypertension and obesity [22,25], which are associated with NCDs [22]. Additionally, work-related requirements play a role in the development and progression of hypertension, for example, long working hours [26], shift work [27], and psychosocial factors [28,29]. Studies in South Africa have reported that NCDs, unhealthy lifestyle factors, hypertension and stressors are prevalent among employees in workplaces [18,30,31,32]. Differences in the prevalence of hypertension by occupational groups have been reported internationally [33]. According to Centers for Disease Control and Prevention (2016), employees with NCDs risk factors, such as hypertension and high cholesterol, cost employers in terms of absenteeism, and lost productivity, than employees with one or none of these risk factors [34].

Workplace may either mitigate or accentuate risk factors for hypertension [35]. Work is also considered as a captive environment in which people can be contacted for recruitment and program implementation [36]. Limited use of the available in-house clinic observed among most employees in this particular Logistics Company, despite the adequate equipment to diagnose and treat hypertension, is alarming. This was apparent during a wellness day organized by the company, when half of the employees recorded high levels of blood pressure not to their knowledge. This could only imply that many of these employees are living with undiagnosed hypertension, which is progressively exposing them to future unfavorable health outcomes, if left untreated. In addition, absenteeism due to ill health has been observed to be high, which might affect the productivity of the company. Researchers have reported that, when logistics performs well, there is trade expansion, export diversification, ability to attract foreign direct investments, and economic growth [37]. Yet data on NCDs is scarce in various companies, including this Logistics Company.

In view of this, the study aimed to determine the prevalence of undiagnosed hypertension and associated risk factors among employees in a Logistics Company, in the Gauteng Province of South Africa. Holistic approach of NCD management at a workplace is strengthened by both employer and employee education and participation, targeting several approaches including risk management and advocating healthy lifestyles as part of a healthy workplace program [38]. The implications of this study may highlight a need for companies without wellness days and in-house clinics to emulate this Logistic Company, to improve the health of employees, especially in relation to NCDs. While this particular company re-strategize and improve workplace health programs and policies for a proper management of NCDs among its employees.

## 2. Materials and Methods

### 2.1. Study Design and Setting

A cross sectional quantitative study was conducted in a Logistics Company from August 2019 to April 2020. The company is situated in Midrand, a business hub of companies in the Gauteng Province of South Africa. According to BusinessTech, Gauteng was the leading financial nerve center and manufacturing hub of SSA, and currently contributes 35% to South Africa’s Gross Domestic Product [39]. The Logistics Company used in this study specializes as a frozen third-party logistics operation servicing the South African food industry. It is a bulk cold storage enterprise, transporting food in refrigerated primary transport to retailers, and has multi-temperature warehousing and distribution.

In this Logistics Company, a wellness program is organized twice a year (at the beginning of the year, and during mid-year) to promote a healthy workforce, and to identify unhealthy lifestyles and health conditions which affect productivity and that help in reducing absenteeism. In addition, it provides employees with strategies to adopt in maintaining healthy behaviors. The company has an in-house clinic, which is commendable. This approach is in line with WHO recommendations of workplaces as healthy settings to promote health, including interventions for NCDs. It is also in accord with the WHO Global Plan of Action on Workers Health 2008–2017, which was adopted at the 60th World Health Assembly [40]. Research shows that the health status of employees directly influences their work behaviour, attendance and job performance. Hence, effective wellness strategies can help alleviate both absenteeism and presentism, and their related costs [41]. Considering a wellness program, an in-house clinic and several occupations with varied job descriptions and shifts, these characteristics warranted use of this company as a case study.

### 2.2. Study Population, Sampling and Sample Size

The total population of employees in this particular Logistics Company is 1650. A representative sample of 312 was obtained following application of the Rao software sample calculator [42], which took into consideration the total population size of 1650 employees, a 5% margin of error and 95% confidence. We used systematic random sampling to select participants from a headcounts list, and to confirm participants as employees of the company, containing the names of all employees, provided by the company’s human resource office.

The study considered 1598 floor employees and 46 managers and supervisors, making up a population of 1644. The study excluded the six (*n* = 6) executive and human resources personnel, operating from the head office situated outside Gauteng, South Africa. During recruitment, employees who had previously been diagnosed with hypertension/chronic diseases, and were on medication at the time of the study, were excluded from the study, as well as female employees who indicated that they were pregnant.

### 2.3. Data Collection Instruments and Procedures

A modified, validated, self-administered WHO STEPwise questionnaire was used for data collection [43]. The information collected included demographics on occupation (i.e., job description and working shifts), age, marital status and education level, as well as smoking and alcohol use, fruit, vegetables and salt consumption, physical activity, family history of hypertension, and physical measurements (i.e., blood pressure and anthropometry) (see Supplementary Material).

The questionnaire was first prepared in English and then translated into the commonest spoken local language, iSiZulu, and validated through content and face validity, and via a pilot study [44]. Independent translators who speak isiZulu as their mother tongue and are conversant with English completed forward and backward translations of the questionnaire. An expert committee approved the final version of the translated questionnaire [44]. To make sure that the translated items retained the same meaning as the original items, and to ensure that there was no confusion regarding the translated questionnaire, a pilot study was conducted to pre-test it and determine its feasibility [44] among 15 employees who did not partake in the main study.

Blood pressure was measured according to the South African Hypertensive Practice Guideline 2014 using an approved, calibrated and validated electronic device attached to an arm cuff. Three readings were taken at two-minute intervals in a quiet room, an initial reading was discarded and the remaining two readings were recorded to take the average. We predicted workplace hypertension as a systolic blood pressure (SBP) ≥ 140 mmHg or a diastolic blood pressure (DBP) ≥ 90 mmHg, or both, and studied the overall prevalence [45].

Weights for employees were measured to the nearest 0.1 kg using a calibrated smart D-quip electronic scale, and heights to the nearest 0.1 cm using a stadiometer, according to the WHO [46]. Three measurements were taken and the average weight (W) and height (H) were recorded. Body mass index (BMI) was calculated as the weight in kilograms divided by the height in meters squared (BMI (kg/m^2^) = weight (kg)/height (m^2^)). Normal BMI is within 18.5 to 24.99 kg/m^2^. Underweight is defined as BMI < 18.5 kg/m^2^, overweight as BMI of 25 to 29.99 kg/m^2^ and obesity as BMI ≥ 30 kg/m^2^. Waist circumference (WC) and hip circumferences (HC) were measured using a non-stretchable plastic tape measure. Central obesity was defined as WC ≥ 94 centimeters (cm) for males and ≥80 cm for females. Abdominal obesity was defined at waist-to-hip ratio (WHR) more than 0.90 for males and 0.85 for females whilst waist-to-height ratio (WHtR) of 0.5 was used for both sexes [47,48].

### 2.4. Data Analysis

Descriptive and inferential statistics were performed using STATA 14 (StataCorp. 2015, Stata Statistical Software: Release 14, College Station, TX, USA). The interactive effect of undiagnosed hypertension and occupation, as well as other demographics, overweight/obesity indicators and lifestyle variables, were performed using a Chi-square test/Fisher’s exact test. Further relationships of undiagnosed hypertension and selected variables were made through univariate, and multivariate (i.e., independent of occupation) logistic regression analyses. The purposeful selection process began with a univariate analysis of each variable, and any variable having a significant univariate test at *p*-value < 0.2 was selected as a candidate for the multivariate analysis. Adjusted odds ratios (AOR) with a 95% confidence interval (CI) were generated and used to determine the independent strength of the relationship. Significance was considered at *p* < 0.05.

### 2.5. Ethical Consideration

Ethical clearance was granted by the Sefako Makgatho Health Sciences University Research and Ethics Committee, South Africa (SMUREC/H/46/2019: PG). Further permission to conduct the study was obtained from the clinic manager of the Logistic Company, as the clinic is responsible and accountable for employee’s health and the company requested that its permission be issued.

## 3. Results

### 3.1. Characteristics of Employees

Three hundred and twelve (312) employees participated in the study, and the response rate was 100%. Employees were divided into two age groups; <30 years and ≥30 years. Mean age for employees was 40 ± 10 years with a minimum age of 21 years and a maximum age of 68 years. Male employees (82%) were more than females (18%), and predominantly black (91%). Over half (167; 54%) were single while 45% (*n* = 140) were married. Almost half of the employees had post matric education (*n* = 152 (49%)). Some employees smoked (37% (*n* = 114)) and some were alcohol users (38% (*n* = 195)). Salt intake was common among employees at 99% (*n* = 309), while 97% reported consuming fruits and vegetables. Physical activeness was reported in 44% (*n* = 138) of the employees, while 41% came from a family with a history of hypertension (Table 1).

The overall means for SBP and DBP were 138 ± 14.6 mmHg and 85 ± 10.5 mmHg, respectively. In Table 2, both males (49%) and females (53%) had an almost similar prevalence of hypertension. Older employees were significantly hypertensive (49%) compared to younger. Obesity by BMI was significantly higher among females than males (49% vs. 23%), as was central obesity by WC (86% vs. 37%). However, WHR was higher among males than females (77% vs. 24%). The WHtR was significantly high risk among females (84%) compared to males (68%). Older employees were significantly hypertensive (49%) compared to younger. The prevalence of abdominal obesity by WHtR was significantly high among employees aged ≥30 years compared to those aged <30 years.

### 3.2. Occupation Profile

Occupations of employees in this company ranged from managerial (group 1), administration (group 2), checker (group 3), cycle counter (group 4), general workers (group 5—including fork-lifters, reach truck drivers, pickers, mechanics and route tracker), and truck drivers and van assistants (group 6), and were grouped based on link to job description. Most of the employees were in group 6, truck drivers and van assistants (18 (38%)), and group 5, general workers (92 (30%)). The prevalence of hypertension was high among general workers (27%) and truck drivers with van assistants (43%). However, no significant association was observed between occupation and hypertension (Fisher’s exact; *p* = 0.266) (Table 3).

### 3.3. Factors Associated with Hypertension

Table 4 shows comparison of demographic and hypertension status. Undiagnosed hypertension was associated with age, marital status, education, alcohol use and physical activity.

In the unadjusted model at a *p*-value cut-off point of <0.20, age (R = 2.6, 95CI; 1.47–4.63), and marital status (married, OR = 2.0, 95%CI; 1.28–3.18) were associated with hypertension. Hypertension was also associated with smoking (OR = 0.7, 95%CI; 0.45–1.14), alcohol use (OR = 1.7, 95%CI; 1.06–2.68), and physical activity (OR = 0.5, 95%CI; 0.34–0.84). Hypertension was associated with anthropometry, BMI (overweight, OR = 10.8, 95%CI; 1.31–89.3), obesity (OR = 19.5, 95%CI; 2.32–164.3), WC (OR = 4.2, 95%CI; 2.66–6.89), WHR (OR = 2.3, 95%CI; 1.40–3.66) and WHtR [OR =6.6, 95%CI; 3.67–11.78]. WHtR was associated with truck drivers and van assistants (OR = 3.3, 95%CI; 1.16–9.45) and general workers (OR = 2.4, 96%CI; 0.83–6.86). No association was observed between undiagnosed hypertension and occupation (results not shown).

A multivariate model was built using the covariates (i.e., occupation, marital status, smoking, alcohol use, physical activity, BMI (body mass index), and WHR) mentioned-above and results showed that the odds of having hypertension were 2.3 times higher for employees aged ≥30 years (AOR = 2.3, 95%CI; 1.21–4.27). Alcohol use predisposed employees to hypertension almost twice as much as those who did not use alcohol. The odds of hypertension were 2.3 times higher for employees with WC above normal (AOR = 2.3, 95%CI; 1.29–4.07) and those with a WHtR ≥ 0.5 (AOR = 3.7, 95%CI; 1.85–7.30) (Table 5).

## 4. Discussion

This study determined the prevalence of undiagnosed hypertension and associated risk factors among employees in a Logistics Company in South Africa. The overall prevalence of undiagnosed hypertension (50%) recorded among employees in this study is high. This prevalence is almost similar to the 52% reported among a working population in South Africa [18], but higher compared to prevalence reported among workforces in other countries, such as Brazil (32%) [49] and Nigeria (20.1%) [50]. A South African study on hypertension among primary health care professional nurses has reported that 41% of the health care workers were unaware of their hypertensive status [18]. SANHANES has reported that, among those with hypertension in South Africa, 48.7% were not screened, while 23.1% were screened but undiagnosed [21]. More concerning is undiagnosed, untreated, or inadequately treated hypertension, contributing to CVD mortality [5]. Numerous studies have provided evidence of the extent of the unmet need for hypertension services, including the proportion of those with hypertension who are unscreened, undiagnosed, untreated and uncontrolled in Africa, including South Africa [5,21,51,52].

Our data did not show a significant association between occupation and hypertension. The null finding was consistent with the majority of cross-sectional studies on blood pressure and work factors [28]. Significant associations with occupation are more likely to be observed in prospective and ambulatory BP studies [28,35,53]. However, the current study showed that truck drivers, van assistants (3.3 times) and general workers (2.7 times) were more likely to have increased WHtR (unadjusted), compared to employees in other occupations. WHtR is a better discriminator of CVDs than other anthropometric indicators, and it also indicates abdominal obesity [48,54]. In fact, the prevalence of hypertension among truck drivers and van assistants was 43%, and 27% among general workers, which was higher than in other occupations, although not significant. A 45.2% prevalence of hypertension has been reported among truck drivers in a developing country (i.e., Brazil) [55], while a higher prevalence of 57% was reported among taxi drivers [56] and 36% among truck drivers [57] in South Africa. Truck drivers are associated with living and working conditions which increased risk for CVDs [58].

Most of the employees in the current study reported consuming fruits and vegetables, and more than half consumed salt and were physically inactive, while at least a quarter were current smokers and alcohol users. Hypertension is typically attributed to increased urbanization and population aging, along with behavioral risk factors, including tobacco and alcohol use, poor diet and physical inactivity [51]. The consumption of fruits and vegetables by almost all employees is inconsistent with findings on low consumption of fruits and vegetables reported in South Africa [32,59]. High salt consumption is also a key driver of hypertension, and there is strong evidence to indicate that South Africans consume up to two to three times the recommended daily allowance of five grams [60]. This is supported by the South Africa Demographic and Health Survey (SADHS), which has reported unhealthy eating in the population [61]. Two thirds of the workforce has been reported to be physically inactive in the current study, similar to other South African studies [32,61]. On the other hand, a quarter of employees in the current study reported current smoking, which is higher than previous research in the workforce (26%) [32].

The current study recorded 38% alcohol use compared to 25% [62] and 29% [32] reported in industry in South Africa. Apparently, South Africa has one of the highest risky drinking patterns in the world [63], proven by several studies conducted in various population groups [64,65,66]. Amongst employees, rates of harmful alcohol use are high in several workforces in South Africa, such as the public, industrial and financial sectors [67]. Evidence has found that alcohol, and in particular heavy drinking, increases the risk of unemployment, and for those employed it increases absenteeism [68]. Our data showed that alcohol use predisposed employees to hypertension almost twice as much as those who did not use alcohol. The Prospective Urban and Rural Epidemiology (PURE) study in South Africa showed that employees self-reporting the consumption of alcohol had a 30% increased risk of developing hypertension [69]. This higher level of both hypertension and alcohol use may be explained by the threshold effect, where alcohol consumption exacerbates physiological damages that may lead to hypertension [70].

In this study, the odds of having hypertension were 2.3 times higher for employees aged ≥30 years. This is similar to other studies that have reported the prevalence of hypertension increasing with age among the working population in developing and developed countries [71,72]. The association between age and hypertension has been documented in several African studies [51,73,74]. A possible mechanism of age-related hypertension entails the fact that age induces an increase in visceral fat and circulating leptin, which is associated with a significant increase in blood pressure [75]. Literature documents that metabolic syndrome promotes arterial stiffening and accelerates vascular aging and development of hypertension in humans [76,77,78]. This has also been suggested among health care workers as they age [18].

More than half of the employees in this study exceeded the risk thresholds for overweight/obesity. Obesity in the workforce is associated with difficulty performing work in confined spaces, decreased productivity, greater absenteeism, higher turnover and cost to company [61]. In addition, obesity has been associated with higher odds of hypertension among employees in developed [79] and developing countries [80,81]. The odds of hypertension were 2.3 times higher for employees with abdominal obesity (by WC) and 3.7 times higher for employees (by WHtR) in this study compared to employees with normal WC and WHtR. It is worth noting that overweight/obesity is one of the main public health concerns in South Africa among various population groups [82,83,84,85]. When obesity coexists with hypertension, it may further increase the development of CVDs [71]. The sympathetic nervous system over-activation, stimulation of the renin–angiotensin–aldosterone system, alterations in adipose-derived cytokines, insulin resistance, and structural and functional renal changes are implicated in obesity–hypertension complex [86].

These findings emphasize a need for intensified health promotion and NCD prevention, and Schouw et al. [32] have reported that the workplace is ideally suited for targeted interventions. Strategies to prevent hypertension could include intervention at the company level, promoting staff canteens, even in small and medium-sized companies, health promotion programs [36] and dietary modification interventions [87]. At the individual level, strategies could include interventions to control alcohol use and education about healthy eating [88]. The implementation of joint interventions would probably be more effective [87], particularly if targeting a well-defined population. As a result, the work environment directly shapes employee health and health behaviors, and acts as an accelerator or preventer of chronic disease [89].

## 5. Limitations and Strengths

It should be noted that this study has several limitations and strengths. A strength of this study is that it has scientifically quantified, and provided evidence on, undiagnosed hypertension and risk factors in the case of a Logistics Company in Gauteng, considering the presence of the in-house clinic and the two wellness programs taking place annually in the company, useful for further intervention. A second strength is that the survey measures were collected via self-reporting, resulting in very little missing data in the final analysis models, although the method is subject to recall errors. A third strength is that multilevel adjustment for covariates, which indicates the generalizability of the study results to companies with a similar environment and working conditions, is likely to be reliable. In terms of limitations, first, the data came from a cross-sectional survey in a homogenous working group at a Logistics Company used as a case study, which would not be representative of the entire working population in various companies in South Africa. Thus, the generalizability of current reported information to other regions should be limited. Second, the design of the cross-sectional study leaves open for interpretation the casual relationships between hypertension and their risk factors, which implies that our results must be interpreted with some caution. Third, many of the findings were self-reported such as tobacco and alcohol use, fruits, vegetables and salt consumption, which could lead to bias, in the sense that employees might have under-reported; hence, these variables should be interpreted with caution. In addition, fruits, vegetables and alcohol consumption, as well as physical activity, might have been under-estimated or under reported due to the social desirability of answering questions and the dichotomous questions used. Finally, our results lack biochemical markers, such as lipids, insulin and glucose, to accompany the physical measurements reported in this study, which would add value, and should be considered in the future. Prospective cohort studies are needed to determine the extent of the associations of demographic and lifestyle factors with hypertension mediated by occupation in various companies in the trading industry.

## 6. Conclusions

A high prevalence of undiagnosed hypertension was observed among the employees of this Logistics Company. Associations with hypertension ranged from factors such as age, marital status, education, alcohol use, physical activity, BMI (i.e., overweight and obesity), WC and WHtR (i.e., abdominal obesity) among employees. Our study findings contribute to the limited scientific literature on the association between undiagnosed hypertension and risk factors from the perspective of a logistics workplace. The findings also indicate that, among all job types, truck drivers and van assistants were more affected by undiagnosed hypertension and had a higher prevalence of abdominal obesity. A detailed subpopulation analysis to target high-risk occupations and an assessment of the interaction between significant risk factors are recommended. Future longitudinal studies are needed to determine the mediation of occupation, in its associations with demographic and lifestyle factors, with hypertension in various workplaces. Improved and effective workplace health programs and policies are necessary to contribute to proper management of NCDs, especially undiagnosed hypertension, among employees.

## Figures and Tables

**Table 1:** Selected demographic variables and lifestyle factors.

Variables	Categories	Frequency	Percentages (%)
Age	<30 years	66	21
	≥30 years	246	79
Gender	Male	255	82
	Female	57	18
Race	Asian	8	3
	Black	284	91
	Colored	3	1
	White	17	5
Marital status	Ever married	140	45
	Single	167	54
	Divorced	5	16
Level of education	Primary school	2	0.6
	Secondary school	37	12
	Matric	121	39
	Post matric	152	49
Smoking	No	198	64
	yes	114	37
Alcohol use	No	117	63
	Yes	195	38
Salt intake	No	85	24
	Yes	265	76
Fruits consumption	No	10	3
	Yes	302	97
Vegetables consumption	No	8	3
	Yes	304	97
Physical active	No	174	56
	Yes	138	44
Diagnosed with Diabetes	No	292	94
	Yes	20	6
Family history of hypertension	No	184	59
	Yes	128	41

**Table 2:** Comparison of prevalence of hypertension and selected variables by gender and age.

Variables	All	Females	Males		<30 Years	≥30 Years	
	*n* (%)	*n* (%)	*n* (%)	*p*-Value	*n* (%)	*n* (%)	*p*-Value
Blood pressure							
Non-hypertensive	156 (50)	27 (47)	129 (51)	0.660	45 (68)	129 (51)	0.001 *
Hypertensive	156 (50)	30 (53)	126 (49)		21 (32)	126 (49)	
BMI (kg/m^2^)							
Underweight	9 (3)	2 (4)	7 (3)	0.001 **	2 (3)	7 (3)	0.135 **
Normal	97 (31)	13 (23)	84 (33)		27 (41)	70 (28)	
Overweight	120 (39)	14 (25)	106 (42)		25 (38)	95 (37)	
Obese	86 (28)	28 (49)	58 (23)		12 (18)	74 (30)	
WC							
Normal	168 (54)	8 (14)	160 (63)	≤0.0001 *	42 (64)	126 (51)	0.072
Central obesity	144 (46)	49 (86)	95 (37)		24 (36)	120 (45)	
WHR							
Normal	206 (66)	13 (23)	193 (76)	≤0.0001 *	50 (76)	156 (63)	0.060
Abdominal obesity	106 (34)	44 (77)	62 (24)		16 (24)	90 (37)	
WHtR							
Normal	90 (29)	9 (16)	81 (32)	0.016 *	28 (42)	62 (25)	0.006 *
Abdominal obesity	222 (71)	48 (84)	174 (68)		38 (58)	184 (75)	

*—indicates a significant association by Chi2, **—indicates significant association by Fisher’s exact, BMI = body mass index, WC = waist circumference, WHR = waist hip ratio, WHtR = waist to height ratio.

**Table 3:** Occupation profile of employees.

Groups of Occupations	*n*	Hypertensive *n* (%)	Non Hypertensive *n* (%)	Working Shifts	Summarized Job Description
Group 1 (Manager)	17	9 (16)	8 (5)	Day shift:	Processing orders, Operating mechanical and IT systems, Liaising with transport companies, suppliers and clients, etc.
				7 a.m. to 4:30 p.m.	
				Night shift:	
				5 p.m. to 3 a.m.	
Group 2 (Administrator)	41	22 (14)	19 (12)	Day shift:	Receiving, checking and scanning of all incoming stock and shipments, Responsible for coordinating Exports shipments to suppliers, etc.
				7 a.m. to 4:30 p.m.	
				Night shift:	
				5 p.m. to 3 a.m.	
Group 3 (Checker)	32	12 (8)	20 (13)	Day shift:	Checks, processes, and clears customers’ orders for shipments, Keeps the warehouse and its environs clean to ensure safety, Operates, cleans, and maintains all the equipment used in the warehouse, etc.
				7 a.m. to 4:30 p.m.	
Group 4 (Cycle counter)	12	4 (3)	8 (5)	Morning shift:	Perform daily cycle counts, Monitoring and controlling inventory integrity, Maintain product identification, location, and lot code accuracy, Open cartons, bundles, and other containers to count items and/or weigh materials to determine quantity on hand, etc.
				3 a.m. to 1 p.m.	
Group 5 (General workers: including fork lifters, reach truck drivers, mechanics, route tracker and picker) Mechanic Route tracker Picker	92	42 (27)	50 (32)	Day shift:	Unload and upload material, Identify damages, Report shortages, Report quality deficiencies, Transport raw materials to production workstations, Inspect machinery, Determine the need for repairs, Keep updated records of inventory, Produce activity logs Checking and fixing trucks, machinery and forklifts and reach trucks.
				6 a.m. to 5 p.m., or until the delivery is done	
				Night shift:	
				5 p.m. to 3 a.m.	
				Day shift:	
				7 a.m. to 4:30 p.m.	Dispatching of trucks in the morning, Monitoring Drivers and Van-assistant time keeping achieving scheduled departure times for all routes, etc.
				Day shift:	
				5 a.m. to 5 p.m.	
				Night shift:	Manages pick ticket orders, Pulls warehouse items from the shelves based on number, size, color, quantity, and quality requirements, Ensures that orders are accurate, Operates handling equipment
				5 p.m. to 5 a.m.	
				Day shift:	
				7 a.m. to 4:30 p.m.	
				Night shift:	Shrink wraps products to pallets, Loads delivery vehicles, etc.
				5 p.m. to 3 a.m.	
Group 6	118	67 (43)	51 (33)	Day shift:	Inspect truck for defects and safe operating condition before, during and after trips, Drive truck to and from designated destinations, etc.
(Includes truck drivers and van assistants)				6 a.m. to 5 p.m., or until the delivery is done	Loading and unloading company products, Assisting drivers to find delivery locations, etc.
				Night shift: 5 p.m. to 3 a.m.	

**Table 4:** Comparison of selected variables by hypertension status.

Variables	Normal	Hypertensive	*p*-Values
Age (years)			
<30	45 (29)	21 (13)	0.001 *
≥30	111 (71)	135 (87)	
Gender			
Female	27 (17)	30 (19)	0.660
Male	129 (83)	126 (81)	
Marital status			
Ever married	57 (37)	83 (53)	0.005 **
Single	97 (62)	70 (45)	
Divorced	2 (1)	3 (2)	
Level of education			
Primary school	1 (0.6)	1 (0.6)	0.048 **
High school	11 (7)	26 (17)	
Matric	64 (41)	57 (37)	
Post matric	80 (51)	72 (46)	
Smoking			0.158
No	93 (60)	105 (67)	
Yes	63 (40)	51 (33)	
BMI (kg/m^2^)			≤0.0001 **
Normal	72 (46)	25 (16)	
Underweight	8 (5)	1 (1)	
Overweight	51 (33)	69 (44)	
Obese	25 (16)	61 (39)	
Alcohol use			0.026 *
No	68 (44)	49 (31)	
Yes	88 (56)	107 (69)	
Salt intake			1.000
No	2 (1)	1 (0.6)	
Yes	154 (99)	155 (99.4)	
Fruits consumption			1.000
No	5 (3)	5 (3)	
Yes	151 (97)	151 (97)	
Vegetables consumption			1.000
No	4 (3)	4 (3)	
Yes	152 (97)	152 (97)	
Physical activity			0.006 *
No	75 (48)	99 (63)	
Yes	81 (52)	57 (37)	
Diagnosed with Diabetes			0.165
No	149 (96)	143 (92)	
Yes	7 (4)	3 (8)	
Family history of hypertension			0.818
No	93 (60)	91 (58)	
Yes	63 (40)	65 (42)	

*—indicates a significant association by Chi2, **—indicates significant association by Fisher’s exact, BMI = body mass index.

**Table 5:** Association of undiagnosed hypertension with covariates.

Variables	AOR	95%CI	*p*-Value
Undiagnosed Hypertension			
Age			
<30 years	1 (Reference)		
≥30 years	2.3	1.21–4.27	0.001 *
Alcohol use			
No	1 (Reference)		
Yes	1.8	1.05–2.93	0.032 *
WC			
Normal	1 (Reference)		
Abdominal obesity	2.3	1.29–4.07	0.005 *
WHtR			
Normal	1 (Reference)		
Abdominal obesity	3.7	1.85–7.30	≤0.0001 *

*—indicates a significant association, WC = waist circumference, WHR = waist hip ratio, WHtR = waist to height ratio. Covariates adjusted for: occupation, marital status, smoking, alcohol use, physical activity, BMI (body mass index) and WHR.

## Data Availability

The dataset for participants generated and analysed during the current study is available from the corresponding author upon reasonable request.

## Supplementary Materials

The following is available online at https://www.mdpi.com/article/10.3390/healthcare9080964/s1. Questionnaire: Undiagnosed Hypertension in a Workplace: A Case of a Logistic Company in Gauteng, South Africa.
