# Supplementary material for: Undiagnosed Hypertension in a Workplace: The Case of a Logistics Company in Gauteng, South Africa
Source: Healthcare (Basel). 2021 Jul 30;9(8):964. doi: 10.3390/healthcare9080964 (PMC8394589; doi:10.3390/healthcare9080964)
Supplement: Supplementary file 1 [file healthcare-09-00964-s001.zip › healthcare-1288326-supplementary.pdf]

healthcare

MDPI

OUESTIONNAIRE

PROJECT TITLE

Undiagnosed Hypertension in a Workplace:
A Case of a Logistic Company in Gauteng, South Africa

Greetings to employees,

"Thank you for agreeing to participate in this study. This study is about undiagnosed hypertension and risk factors among employees in your company. The questionnaire is divided into three sections, namely demographic information, behavioural information and physical measurements. We are starting with measuring weight, height, waist and hip circumferences. Thereafter, we request that you fill the answers for the demographic and behavioural information. If there is a question you do not understand, please feel free to ask Mrs Bokaba. She is available to guide you when you need help".

DEMOGRAPHIC INFORMATION

CORE: Demographic Information		
**Question**		**Response**
1.	Gender	Male ☐<br>Female ☐
2.	What is your date of birth?	Day ☐ Month ☐ Year ☐
3.	How old are you?	Years ☐
**EXPANDED: Demographic Information**		
4.	What is the highest level of education you have completed?	Primary school ☐<br>Secondary school ☐<br>Matric ☐<br>Post matric ☐
5.	What is your racial group?	Asian ☐<br>Black ☐<br>Coloured ☐<br>White ☐
6.	What is your marital status?	Single ☐<br>Ever Married ☐<br>Divorced ☐
7.	What is your occupation?	☐
8.	What is your job description, briefly?	
9.	What is/are your working shift(s)?	☐

BEHAVIOURAL MEASUREMENTS		
**Question**		**Response**
10.	Do you currently smoke?	Yes ☐<br>No ☐
11.	Do you currently consume alcohol?	Yes ☐<br>No ☐

CORE: Diet		
The next questions ask about the fruits and vegetables that you usually eat. I have a nutrition card here that shows you some examples of local fruits and vegetables. Each picture represents the size of a serving. As you answer these questions, please think of a typical week in the last year.		
**Question**		**Response**
12.	Do you **eat fruits**?	Yes ☐<br>No ☐

13.	Do you **eat vegetables?**	Yes ☐<br>No ☐
14.	Do you **eat salt** in your food?	Yes ☐<br>No ☐

CORE: Physical activities

I am going to ask you about the time you spend doing different types of physical activity in a typical week

Question		Response
15.	Do you engage in physical activities?	Yes ☐<br>No ☐

CORE: History of Diabetes and Family history of hypertension

Question		Response
16	Have you ever been told by a doctor or other health worker that you have raised blood sugar or diabetes?	Yes ☐<br>No ☐
17	Is any family member diagnosed with hypertension?	Yes ☐<br>No ☐

PHYSICAL MEASUREMENTS

CORE: Height and Weight

Question		Response
18.	Height	Centimetres (cm) ☐
19.	Weight	Kilograms (kg) ☐

CORE: Waist and hip circumferences

20.	Waist circumference	Centimetres(cm) ☐
21.	Hip circumference	Centimetres(cm) ☐

CORE: Blood Pressure

22.	Reading 1	Systolic (mmHg) ☐
		Diastolic (mmHg) ☐
23.	Reading 2	Systolic (mmHg) ☐
		Diastolic (mmHg) ☐
24.	Reading 3	Systolic (mmHg) ☐
		Diastolic (mmHg) ☐

THANK YOU FOR PARTICIPATING IN THE STUDY!
